# “The satisfaction we get by doing this, you do not get it with any money”: Experiences, and motivations of, and strategies used by community volunteers delivering social or health interventions in India

**DOI:** 10.1371/journal.pgph.0006733

**Published:** 2026-07-09

**Authors:** Brian Zhou, Yashi Gandhi, Miriam Sequeira, Daisy R. Singla, Richard Velleman, Vikram Patel, Shanu Usgaonkar, Seema Sambari, Subhash Pednekar, Pranali Kundaikar, Lalan Karapurkar, Chunling Lu, Abhijit Nadkarni

**Affiliations:** 1 Department of Psychiatry, Yale School of Medicine, New Haven, Connecticut, United States of America; 2 Department of Anthropology, Harvard University, Cambridge, Massachusetts, United States of America; 3 Centre for Global Mental Health, Department of Population Health, London School of Hygiene & Tropical Medicine, London, United Kingdom; 4 Addictions and Related Research Group, Sangath, Goa, India; 5 Campbell Family Mental Health Research Institute, Centre for Addiction and Mental Health, Toronto, Canada; 6 Lunenfeld-Tanenbaum Research Institute, Sinai Health, Toronto, Canada; 7 Department of Psychiatry, Temerty Faculty of Medicine, University of Toronto, Toronto, Canada; 8 Department of Psychology, University of Bath, Bath, United Kingdom; 9 Department of Global Health and Social Medicine, Harvard Medical School, Boston, Massachusetts, United States of America; Johns Hopkins University Bloomberg School of Public Health, UNITED STATES OF AMERICA

## Abstract

While community volunteers are increasingly recognized for addressing supply-side gaps in healthcare delivery (e.g., provider shortages), their role in mitigating demand-side barriers (e.g., stigma) remains underexplored. This qualitative study investigated the experiences, knowledge, and roles of community volunteers in Goa, India. We examined barriers and facilitators to community-based work, and strategies for integrating community participation and outreach in demand-side interventions. We conducted semi-structured, in-depth interviews with 35 community volunteers who deliver health and socio-developmental programs (e.g., “social workers”, village council members, lay health providers). Thematic analysis revealed that volunteers’ work encompassed demand-related tasks such as connecting community members to government welfare schemes, organizing educational events, and providing informational or material resources via contextually-relevant, relationship-driven strategies, such as leveraging influential community leaders and social connections (including via WhatsApp). Participants demonstrated awareness of the social origins of distress (e.g., unemployment), especially post-COVID-19, and expressed willingness to vertically integrate mental health in their work. Facilitators included social recognition, personal satisfaction, and family and political backing. Lack of community support and resources (e.g., insufficient government funding) were significant barriers in continuation of work for community volunteers. These findings suggest the potential of community volunteers as demand-side change agents for mental health interventions, underscoring the necessity of integrating local knowledge, social networks, and non-monetary community-based incentives into scalable global health programs.

## Introduction

Community-based health interventions have become a cornerstone of healthcare delivery, particularly for overcoming health worker shortages in resource-constrained settings. A robust and growing body of literature supports the efficacy of community-based task-sharing models, which redistribute service responsibilities (e.g., screening and identification, providing psychotherapy) from specialists to less highly trained individuals [1–3], including lay or ‘non-specialist’ healthcare providers, community health workers (CHWs), frontline workers, and community health volunteers [4]. In many low- and middle-income countries (LMICs), primary healthcare interventions delivered by community health workers have been instrumental in improving health system performance, addressing health inequities, and facilitating access to essential treatments [5,6]. This study focuses on community volunteers, defined as individuals delivering a community health-related service who do not receive a regular salary and/or hold a formal position within the health system [7].

Many community-based interventions focus on supply-side barriers (e.g., shortage of treatment providers) to address the treatment gap for global health problems [8]. Demand-side barriers, defined as individual, household, or community-level constraints (e.g., financial, social, informational, cultural, or psychological) that limit healthcare utilization independent of service availability, contribute to significant care gaps and warrant further research [9]. Social stigma, misperceptions about mental health problems, and perceived ineffectiveness or mistrust of available care are common barriers to help-seeking and treatment engagement [10–12]. Addressing both supply-side limitations via task-sharing and demand-side barriers via community integration is crucial for scaling up mental health interventions in LMICs such as India [5].

While most studies have focused on CHWs and individuals formally affiliated with health systems, preliminary evidence suggests that community volunteers can mitigate these demand-side constraints by raising mental health awareness, facilitating identification of individuals needing care, providing emotional support and referrals, destigmatizing mental health problems, and ensuring follow-up [7,13]. In other words, demand-side interventions are crucial as they go beyond health promotion interventions that primarily focus on building knowledge to encourage change in health behavior. Innovative programs, such as proactive community case-detection in Nepal [14] and the Vidarbha Stress and Health Program (VISHRAM) intervention in Maharashtra, India [15], demonstrate the impact of community volunteers in promoting help-seeking and treatment adherence. In India, where the mental health treatment gap remains as high as 85% [16], community support is essential for mental health service delivery and patient recovery, particularly given family and social stigma [17,18]. Further studies of the barriers and facilitators that volunteers face in performing community-based work are necessary to facilitate successful implementation and uptake of demand-side health interventions [19]. Although the importance of community integration, social networks, and personal relationships may be intuitively understood in the practical work of community volunteers, their motives to engage and perceptions about incentives remain comparatively underreported in the global literature.

Our qualitative study thus contributes to gaps in literature by investigating the specific tasks, barriers, and facilitators that community volunteers face, particularly when raising awareness and facilitating access to treatment for common mental health problems [20,21]. Goa, the only state in India without Accredited Social Health Activists (ASHAs; women CHWs), presents a unique context for understanding how the roles of community volunteers may differentiate them, in various ways, from CHWs and those who are paid, affiliated with, and accountable to health institutions. This formative research study is embedded within the IMPRESS (*IMP*lementation of evidence based facility and community interventions to reduce the treatment gap for dep*RESS*ion) program, a Hybrid Type 2 Effectiveness Implementation cluster randomized controlled trial being implemented in Goa [22]. The aim of the trial is to test the effectiveness and cost-effectiveness of a volunteer-delivered community intervention in enhancing the demand for and adherence to a counselling for depression program in primary health care settings. As part of the community intervention development, we sought to understand 1) the role and scope of work conducted by existing community volunteers in Goa, 2) barriers and facilitators that community volunteers encountered, 3) their knowledge about mental health and associated services, and 4) strategies for integrating community participation and outreach in demand-side interventions. We sought to understand how our participants’ experiences and strategies can facilitate integration of mental health into their existing work. By exploring the motivations, incentives, and experiences of volunteers involved in supporting community programs, we sought to identify contextually relevant strategies to inform the IMPRESS community intervention.

## Methods

### Study design

A qualitative research study with semi-structured, in-depth interviews was conducted.

### Setting, sample and recruitment strategies

The interviews were conducted in Goa, India, the site of the IMPRESS trial. Goa is a state on the west coast of India with a population of about 1.6 million. Communities are tight-knit and collectivistic; local politics (e.g., panchayat/village councils, self-help groups [SHG], non-governmental organizations, etc.) play a large role in driving change.

Our sample included diverse, community volunteers in existing socio-developmental and health-related programs, independent from the IMPRESS program. We identified participants through a combination of purposive and snowball sampling. Specifically, we first relied on our existing networks (i.e., community volunteers who had collaborated with Sangath in past projects), followed by outreach to youth club leaders, village leaders, and social workers. These local stakeholders connected us to people who have been performing similar community work as the IMPRESS program intended (e.g., arranging community events and engaging with village members for spreading awareness, community building, and/or empowerment). Participants were subject experts in community engagement—these volunteers were already executing the roles and tasks required of community health interventions. Finally, we asked interviewees to connect us with others they may know conducting similar work. We recruited participants until our sample represented a range of geographical areas and socio-demographic characteristics (e.g., formal and informal role in the community, gender, age). Participants were included if they could speak either in English and/or one of the local languages (Hindi, Marathi, Konkani).

### Data collection

The interviews were conducted between 15th May and 30th September 2021. The interview guide explored four key domains: (i) the scope of the current work that the community volunteers are involved in, (ii) the barriers and facilitators they experience in their day-to-day work, and how they navigate these, (iii) the role of incentives in community work, and (iv) their knowledge relating to mental health problems and services, and their willingness to respond to the communities’ mental health needs within their ongoing work. We conducted individual semi-structured interviews with community volunteers, each lasting around 45–60 minutes. The information sheet, consent forms, and interview guides were available in English and all local languages. A socio-demographic form recorded participant data on gender, age, employment status, education, and their role in the community. All interviews were audio recorded with participant consent; most were conducted in-person at a place convenient for the participant, but some were telephonic. The latter was only done when the community volunteers were not comfortable meeting in-person due to the COVID-19 pandemic. The guide was iteratively revised at different time points through the course of the study, based on feedback received about the participants’ understanding of questions and depth of responses across various themes, as captured by summary sheets filled out by interviewers immediately after interviews. These summary sheets were used to ascertain saturation of data, defined as no emergence of new insights of volunteer strategies or suggestions that directly informed intervention development.

All interviews were conducted by one of six field researchers who were from the local region. Four had previous training and experience in conducting community-based interviews. The two new researchers received training in conducting qualitative interviews, supported with practicing via role plays, shadowing and regular supervision. To avoid social desirability of responses, interviewees identified through purposive sampling were interviewed by someone not familiar to them through previous engagement. We ensured that staff who personally knew the participants due to prior engagements were not involved in interviews, to prevent interpersonal power dynamics from influencing participant responses. However, due to the researchers’ affiliation with a non-governmental organization (NGO) conducting projects in collaboration with the health system, it is possible that participants perceived the researchers to have a degree of authority thus influencing their responses.

All field researchers received two training sessions on how to administer the question guide, followed by regular supervision to discuss any challenges and/or feedback on revising the guide. The training and supervision were done by the research coordinator (AG) and research assistant on the project (YG). After the interviews, the audio recordings were transcribed and translated into English by bilingual translators who had nearly five years of experience in the local context. The translator team followed a systematic protocol to ensure quality assurance; it involved reviewing of the transcripts by the Translation Coordinator and double checking in cases where the audio quality was poor.

### Analysis

Two pairs of researchers (AG and YG, BZ and YG) coded the interview data using NVivo 12. In each pair, at least one coder (AG or YG) was based in the local setting and worked closely with field researchers conducting interviews either through training or supervision. AG and YG are Indian researchers, based in Goa, with master’s degrees in Public Health and Global Mental Health, respectively. BZ is a Chinese-American M.D. candidate at Yale School of Medicine. YG’s experience with qualitative research over the past five years, and BZ’s B.A. in Anthropology informed the socially-embedded themes in this analysis. The pairing of coders (with one embedded in the setting and familiar with the socio-cultural context, and the other not) enabled us to engage in critical discussions and reflections about how we were interpreted the data in our regular meetings.

We followed a thematic analysis approach, a widely used methodology to identify patterns and shared meanings across different experiences [23]. Drawing upon Braun and Clarke’s reflexive thematic analysis framework, we used an open coding approach involving a combination of deductive and inductive coding [24,25]. Four researchers (YG, BZ, AG, AN) developed an initial codebook in Excel deductively from the research objectives, interview guide, and initial review of transcripts. Using an open-coding approach, new codes, sub-codes, and emerging themes were inductively added and organized into the codebook. Overarching themes (e.g., barriers and facilitators) were primarily identified deductively based on research objectives, while individual codes and sub-codes were predominantly added and revised in an inductive manner.

**Fig 1:**
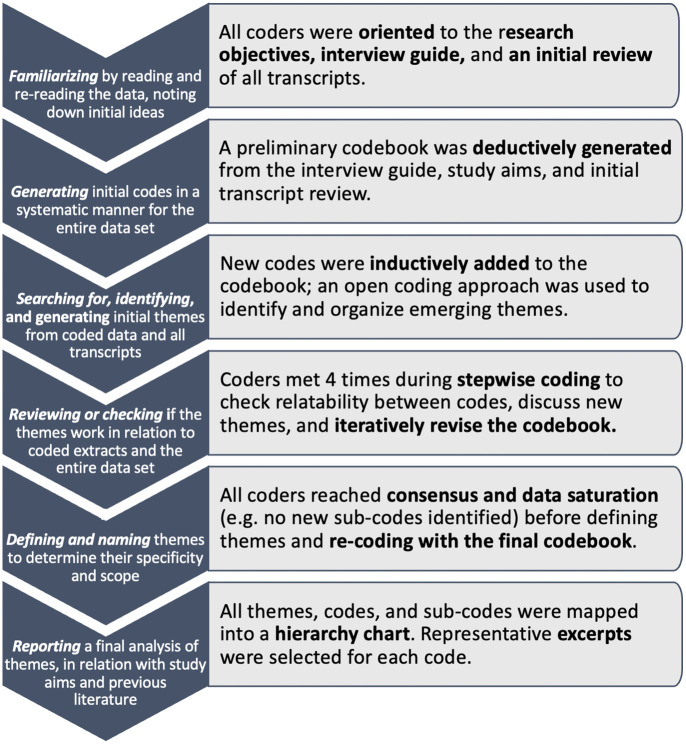
Schematic representation of the reflexive thematic analysis framework and inductive-deductive method used.

Coding was conducted in a stepwise manner and involved two coders meeting a total of four times during the process to facilitate iterative revisions of the codebook. We independently coded five transcripts in the first round, and ten in the subsequent rounds, and then discussed discrepancies between coders until substantial agreement was achieved. At each iteration of the codebook, sub-codes were merged as needed, along with sub-themes into larger themes. All coders reached consensus (e.g., no new subcodes were identified) before re-coding transcripts based on the final codebook as needed. After coding, we organized thematic data into a diagram, cross-checked coding frequency scores between all coders, and selected quotations that exemplified each theme. While we considered the use of an intercoder reliability metric, further review of methods indicated that reliability is not an appropriate criterion for judging qualitative work and that quantitative measures of intercoder reliability assume an epistemologically problematic, positivist standard [26,27]. Thus, we opted not to use or report quantitative metrics to assess qualitative agreement, but alternative measures for data validity and quality were taken, including transparent reporting of analytic procedures, “thick description” with plentiful excerpts of raw data, and supervision of codebook development and coding process by senior researchers (AN, DS, VP). Fig 1 depicts a summary of the reflexive thematic analysis framework and our actions at each stage.

### Ethical considerations

Ethical approvals were obtained from London School of Hygiene and Tropical Medicine (sponsoring organization) and the Institutional Review Board of the local implementing organization, Sangath (reference number: AN_2017_033). All participants individually provided written informed consent. The consent process involved detailed information on the study’s objectives, the specific information gathered in interviews, the voluntary nature of participation, the potential benefits and risks, and how data will be kept anonymous and confidential. All information was provided in written and oral format in the local language (Hindi, Konkani or Marathi). Participants were encouraged to ask clarifying questions during the consenting process.

## Results

A total of 35 community volunteers were interviewed. Sociodemographic details are shown in Table 1.

**Table 1:** Sociodemographic data of participants.

	N = 35	
	n	%
**Socio-demographic characteristics**		
**Gender**		
Male	7	20
Female	28	80
**Area living**		
Rural	28	80
Urban	7	20
**Age (in years)**		
31–40	3	8.5
41–50	16	46
51–60	10	28.5
61 and above	6	17
**Highest educational level**		
Secondary school (5th to 10th grade)	12	34
Higher secondary school (11th to 12th grade)	7	20
Diploma in home nursing after 12th grade	1	3
Undergraduate degree	14	40
Post-graduate degree (Master’s or PhD)	1	3
**Employment status**		
Employed full-time	4	11
Employed part-time	7	20
Self-employed	9	26
Unemployed and currently looking for work	2	6
Unemployed and not currently looking for work	12	34
Retired	1	3
**Formal Occupation**		
Voluntary work (i.e., not working for pay)	26	74.3
Government job	5	14
Community resource person	2	5.7
Clerical worker (e.g., secretary)	1	3
Professional (e.g., engineer or doctor)	1	3
**Volunteer Role**		
Self-help group member or leader	11	31
“Social worker”	9	25.5%
Panchayat (village council) member	5	14
Community resource person	2	5.7%
Block resource person	2	5.7%
Sarpanch (village council president)	2	5.7%
Sakhi (lay mental health provider)	2	5.7%
Anganwadi teacher (rural childcare provider)	2	5.7%

Most participants (n = 28 of 35, 80%) were female and lived in rural parts of Goa. On average, participants were 50.51 years of age (range 31–72). A significant minority had a bachelor’s degree (n = 14, 40%), while 34% completed education until Grade 10, and 20% until Grade 12. Nearly one-third were unemployed (n = 12, 34%) and not currently looking for work, followed by 26% (n = 9) who were self-employed and 20% (n = 7) who were employed part-time. 74.3% (n = 26) engaged in voluntary work (i.e., not working for pay), while 14% (n = 5) held government jobs.

Regardless of formal occupation, participants performed various volunteer roles. Social workers and self-help group members each comprised a quarter (n = 9, 25.5%) of the sample. To clarify, “social workers” interviewed in this study do not have formal degrees in social work; in the local context, volunteer work supporting socio-developmental livelihoods and health-related programs is colloquially referred to as “social work”. Self-help groups (SHG) are informal micro-financing cooperatives of women who pool financial resources for joint socio-economic activities. Another 20% of the sample were village council presidents (‘Sarpanch’, n = 2, 5.7%) and representatives (‘Panchayat’ members [PM] (n = 5, 14%). Four participants served as Community Resource Persons [CRP], (n = 2, 5.7%) or Block Resource Persons [BRP] (n = 2, 5.7%)—they connected community members with resources, sometimes supported health data survey collection, and supported program implementation in rural villages. 11.4% of volunteers provided community health services: two Sakhis delivered lay mental health treatment, and two Anganwadi teachers provided education and primary healthcare in rural childcare centers.

### Role and scope of work

Participants reported a range of community health initiatives that they organized, including educational competitions and programs focused on nutrition, yoga, and children’s health. Most volunteers described working with primary health centers in organizing free “health camps” or *“different programs …* [for] *diabetes check-up, dental check-up, blood test, everything… held in the village”* (CRP, Female, 42 years). Community volunteers reported hosting health programs, events, and competitions in schools, temples, community/village council (*Panchayat*) halls, individual houses, and community-based health or childcare centers (*Anganwadis*).

Participants conducted a wide range of volunteer work, most of which fell into the following categories: connecting community members with existing programs, empowering communities with education or training, organizing events, and providing resources or financial assistance. Understanding the current scope of work for volunteers provided opportunities to assess possible integration of mental health into existing community work.

#### Connecting community members with existing programs.

Over half the participants reported connecting community members to government programs, by assisting with paperwork, liaising with public officials for program delivery, and working with health centers to identify patients. One volunteer described *“explaining all the government schemes… what benefits they would like to avail, what other forms are needed… poor people who do not have access to* [cooking] *gas, I give them those forms and arrange gas for them”* (CRP, Male, 72 years).

Notably, these volunteers went beyond simply referring individuals or sharing information: our participants reported accompanying community members to hospitals or government offices, filling out forms on their behalf, and actively connecting them to many government schemes, ranging from agriculture to economic support. For instance, one participant explained that families received INR 2 lakhs (~2000 USD) if a household member died from COVID-19, and she would help families access government schemes *“and call people department wise”* (SHG President, Female, 57 years). Some community volunteers also provided career or financial advice, facilitated help-seeking, and resolved family or community disputes.

#### Empowering communities via education and training.

Many participants were involved with volunteer or self-help groups, which create opportunities for skill development and economic empowerment. A panchayat member (PM) organized “*courses and training allowing them* [community members] *to run their business… it can be needlework or something else which will help them to earn something”* (PM, Female, 48 years). Other volunteers aided community members in setting up and advertising small businesses. *“We can give them beautician course, bakery course… Or whatever small business that they can do by staying at home… when a woman gets empowered, a house gets empowered”* (PM, Male, 33 years).

#### Organizing events.

A quarter of the participants reported organizing competitions, community celebrations, and religious or cultural events. While most events were not health-related, several volunteers recognized their contributions to mental and social wellbeing: *“In October* (school holidays)*, they* (children) *used to be at home, getting bored, having tension… We used to organize drawing competitions… to entertain them and to keep them happy”* (CRP, Female, 42 years). Holidays, such as Diwali or Shigmotsav (spring festival in Goa), provided opportunities for community gatherings and cultural education. *“We tell the children from today’s generation, if you want to conserve the cultural events… then you have to learn from elders like us”* (PM, Male, 51 years).

#### Providing resources or financial assistance.

Community volunteers organized fundraising and programs to provide necessities and/or financial assistance. “*We gave things to the people who are poor... Rice, lentils... we do our collections, put it in the bank, if anybody needs money, we give*” (Anganwadi teacher, Female, 51 years). During the COVID-19 lockdown, volunteers distributed rations while raising awareness regarding the effects of the virus. For some people who could not afford hospital bills, *“whatever money that has to be collected, I go to our well-wisher friends and tell* [them]*… they give something… or puts in the hospital’s account”* (PM, Male, 33 years).

### Volunteer strategies to facilitate community programs

Community volunteers utilized many strategies to overcome program-related challenges, such as lack of community participation or awareness. Most participants shared that simply providing information about the event details may not suffice; they highlighted the role of reminders, incentives, and personal connection, as key aspects that ensured participation. The perceived effectiveness of various volunteer strategies informed intervention development and community engagement during the trial.

#### Informational strategies.

The most common strategy to inform community members about programs was word-of-mouth, whether through WhatsApp texts, phone calls, group meetings, or door-to-door visits. Participants reported going to “*each and every house*” when necessary to generate awareness for events and programs. *“It is a chain system. There are self-help groups in every village. When you tell the leader of the group, they tell all the group members… there is this training organized at this place… Mostly everybody is familiar... This is how we have done networking with the people and got them closer”* (Social Worker [SW], Female, 63 years).

Social media, particularly WhatsApp, was also a powerful tool to maintain group connection and encourage community participation. Due to COVID-19, some participants utilized WhatsApp to shift training and programming online: *“When we gave ration to 65 families, video of that was made and uploaded in WhatsApp group… by watching that video, many came to ask for ration”* (SW, Female, 45 years). Occasionally, volunteers posted announcements and hosted educational skits in public spaces. *“Main bus stand which is there, like among the youth, they used to act out plays… skits* [on] *mental health problems… it is a different way of approaching people”* (SW, Female, 45 years).

#### Engagement strategies.

Most volunteers emphasized the importance of prior communication of program details, including benefits of attending the program and logistical arrangements. It was typically done through personal contacts or trusted information sources. Many engagement strategies relied upon generating personal buy-in, through rapport building and involving influential persons or community leaders. *“If they have someone they call Chacha* (uncle)*, in their locality, if everybody listens to him, we would make him the chief guest… everybody would come for the program”* (Sakhi [lay mental health worker], Female, 40 years).

Some volunteers reported that financial and non-financial incentives, such as highlighting free health services or organizing competitions with prizes, increased community participation. *“We tell them… instead of going to the Doctor and spending your money, why don’t you all take a free check-up? Now when they organized the first program,* [the] *majority wasn’t there… Sister motivates others nicely by conducting programs, keep prizes because of which they get excited and come for the program. We also do drama shows* [folk theatre/skits]*”* (CRP, Female, 42 years). Volunteers also reported making phone calls or home visits to remind participants to attend programs.

### Barriers and facilitators to scaling-up community interventions

Participants discussed barriers in engaging community members and implementing programs, especially when aiming at reaching a wide audience. Poor and/or misinformed community perception or engagement relating to ‘social work’ and ‘social workers’, lack of financial support from the government, and gender norms and expectations constituted key barriers. Participants shared that active support from community in terms of financial backing or logistical support, a supportive family environment to engage in the unpaid work, and personal contexts (e.g., having personally experienced similar struggles) helped make the work meaningful and motivated them to continue. Identification of barriers and facilitators for sustained volunteer engagement contributed to designs for a hybrid, multi-tiered approach to volunteer incentives, including both financial and non-financial compensation.

#### Community and program-related barriers.

The greatest challenge reported by participants was the lack of community support and understanding of the ‘role’ they play. A general propensity to criticize, undervalue, and question social work made it difficult to engage community members. *“Out of 10, only 2 people appreciate you, and the rest keep* [call] *names… no one tries to understand that there are also difficulties*” (Anganwadi Teacher, Female, 72 years).

Some participants described negative perceptions of NGOs as profit-motivated, alongside stereotypes of volunteers performing social work as a precursor to *“enter politics, everything then becomes dirty*” (SW, Female, 45 years). Participants described lack of resources or government funding for programs, which were exacerbated by the pandemic, as another barrier. Garnering government support posed a challenge, since “*8-10 days get wasted… just to get the permission”* (SW, Male, 43 years).

Scheduling, time management, and lack of family support also affected participation. *“Nobody steps forward because they do not have time. There is no monetary gain in this… some members get shouted at by their families, ‘What benefit do you have?’”* (PM, Female, 42). For program participants, *“the main problem is transport… 80 percent attendance is women… If they come walking… 4 kilometers… it is not possible”* (PM, Male, 32 years).

Furthermore, community volunteers noted gender norms and sexism. For female social workers, family expectations and societal perceptions that *“women should not step out”* restricted their ability to organize. *“The members in our Panchayat were different; they do not support women… They say, “What are these women? Where do they go daily?” But we do not pay attention to them. Because we do it for ourselves”* (SHG Member, Female, 44 years).

#### Facilitators for community programs.

At the community level, a major motivator was local support. One participant discussed donations—a fridge and 200 food packets from neighbors for their program—as a facilitator. Furthermore, several participants emphasized government support and Panchayat members who helped secure resources and space for programs. Community volunteers mentioned financial and political assurance as key supports: *“‘If you want some financial help then Panchayat will be always there for you’, because of that assurance I went forward”* (SHG Member, Female, 44 years).

At the interpersonal level, most community volunteers emphasized the need for family support to be able to perform their work. Some female interviewees described financial and emotional support from their partners, children, and family in balancing community and domestic work. *“My family members never stopped me. Instead, they were the ones who encouraged me to contest the elections… there are some women in the group whose family members ask them, ‘Why are you doing that work?’ Family support is needed”* (PM, Female, 42 years).

At the personal level, half the participants described moral fulfillment and service-oriented values as powerful motivators. According to one volunteer, *“even though I have not taken any education in this field… I had a liking for this right from the beginning. So, there are some like me who work for a moral cause… they get a kind of satisfaction that they have done something for their society”* (Sakhi, Female, 40 years). Other participants described how political aspirations and/or personal struggles gave purpose to their work. *“I had experienced what is poverty… today I go forward by taking that same aim because I do not want other children to face the struggles that I have had”* (SW, Female, 53 years). Several volunteers also cited community blessings as sufficient motivation: *“Because of these people’s well wishes… I feel very happy because... I have done people’s work, they will never forget me”* (SW, Male, 43 years).

#### Incentives for community volunteers.

Most community volunteers refused monetary incentives, although a few accepted honorariums. Almost all volunteers discussed intangible incentives such as personal satisfaction and community appreciation. *“We did social work because of passion… the satisfaction that we get by doing this, you do not get it with any money”* (Anganwadi Teacher, Female, 72 years).

Participants suggested non-financial incentives, including certificates and media recognition (e.g., articles in local newspapers). Several volunteers believed that their social work would be rewarded in spiritual blessings. *“Saying, ‘God bless you’ is a big thing … Not only me but my children also got it. Saying words like ‘God bless you’ is more than enough”* (SHG President, Female, 57 years).

### Community recognition of mental health problems and needs

Participants discussed their understanding of causes and manifestations of mental health problems and where the communities around them may access care. Most agreed on the need for integration of mental health awareness and treatment into existing community programs; almost all indicated willingness to serve as a delivery agent for the IMPRESS program. Participants provided key suggestions on the required training support, the activities they would be willing to support (e.g., planning of meetings with key stakeholders, awareness sessions, accompanying people to health care services), and their expectations relating to incentives and reimbursement. Participant recommendations were incorporated into intervention development to facilitate integration of mental health into existing community frameworks.

#### Mental health awareness.

Community volunteers characterized mental health through various emotions, thought processes, and causal factors. For most participants, the understanding of ‘mental health’ was restricted to ‘mental health problems’; for instance, many characterized depression as an inability to think rationally and control negative thoughts.

While one volunteer stated that mental health problems exist due to genetic causes, most participants cited social causes of distress. *“Mental health is... suppose there is a woman and if the environment in her house is not good, if there is a pressure on her... like her family causes problems for her, or having no children… She keeps quiet. And when it gets too much... she might go into depression”* (SW, Female, 53 years).

In the aftermath of COVID-19, more participants attributed depression to unemployment, poverty, and family hardships, which many experienced during the pandemic. *“Depression means-- during this lockdown... there is unemployment, many are idle so there are arguments among husband-wife… there is no salary… there is future worry… so many cases like this.”* (SW, Female, 45 years).

Furthermore, some participants extended a social causation of mental health to substance use disorders. “*When they do not have money, they go to the bar to drink… and then go home and harass their wife… they think that after drinking alcohol, tension will go, then they get addicted*” (PM, Female, 48 years). Another social worker expressed great concern about *“children normally smoking, consuming alcohol”* due to family, academic, or socioeconomic stress (SW, Male, 32 years).

#### Mental health services and needs.

Most participants expressed the lack of available evidence-based mental health services, information, and awareness. *“Presently, there is nowhere. If you get tensed, you do not have any service as such… there are ayurvedic medicines that have come up… but everyone might not get benefit from it.”* (Sarpanch, Male, 72 years). Many participants expressed a need for counseling services, mental health programs, and meditation centers.

Several participants suggested that hospitals and tertiary care may be one of few options, while others mentioned private doctors and primary health centers. One interviewee referenced a drug addiction program, and six others mentioned mental health awareness, counseling, or meditation camps.

#### Integration of mental health into existing community programs.

Over half of the participants indicated their willingness to be involved as a delivery agent in a mental health program. When asked about integrating mental health awareness with existing community programs, several volunteers referenced their experience with government schemes, such as Bima Yojna (agricultural scheme) and the Anganwadi system. One participant discussed applying engagement strategies from their existing work to mental health interventions: “*If it is through health [Directorate of Health Services], our women are ready, we can call, now if we share messages then women get together… some do not know whether there are doctor for counselling or no… if you take hall in Panchayat then our women can come*” (CRP, Female, 72 years).

Most volunteers indicated their willingness to help with awareness generation, meetings/logistics, and accompanying patients. *“I can give you all the help possible as a volunteer. While going somewhere, if I come across anybody who is really in need of it, then I will contact you… I will take them there and get their check-up done.”* (Sarpanch, Female, 53 years). Almost all participants discussed their available time, ranging from a commitment of four hours to four days per week.

Multiple participants emphasized the importance of knowledge, training, and curriculum materials around mental health. Two participants inquired about online training and indicated general familiarity with virtual meetings. In contrast, another participant stated, “*when we speak face to face, your expressions we can see… the contact increases that’s why it’s better to speak offline*” (SW, Female, 50 years).

Regarding their involvement in a mental health program, half the participants reported no expectations of monetary incentives, while the other half expressed desire for an honorarium or stipend. “*I do not have any this [expectation] with regards to money… but when you spend your day, you waste your petrol and do one work… and if something is given, I don’t say no, I will take… it cannot be totally free*” (SHG President, Female, 54 years).

Many participants also discussed reimbursements for travel, data usage, and food to mitigate financial barriers. *“Every time they cannot ask money from home, from husband… so, they should get something. Because all are not financially well… capable”* (SHG Resource Chair, Female, 51 years).

Overall, community volunteers recognized the importance of addressing mental health and integrating awareness generation, accompaniment, and program implementation in their work.

**Fig 2:**
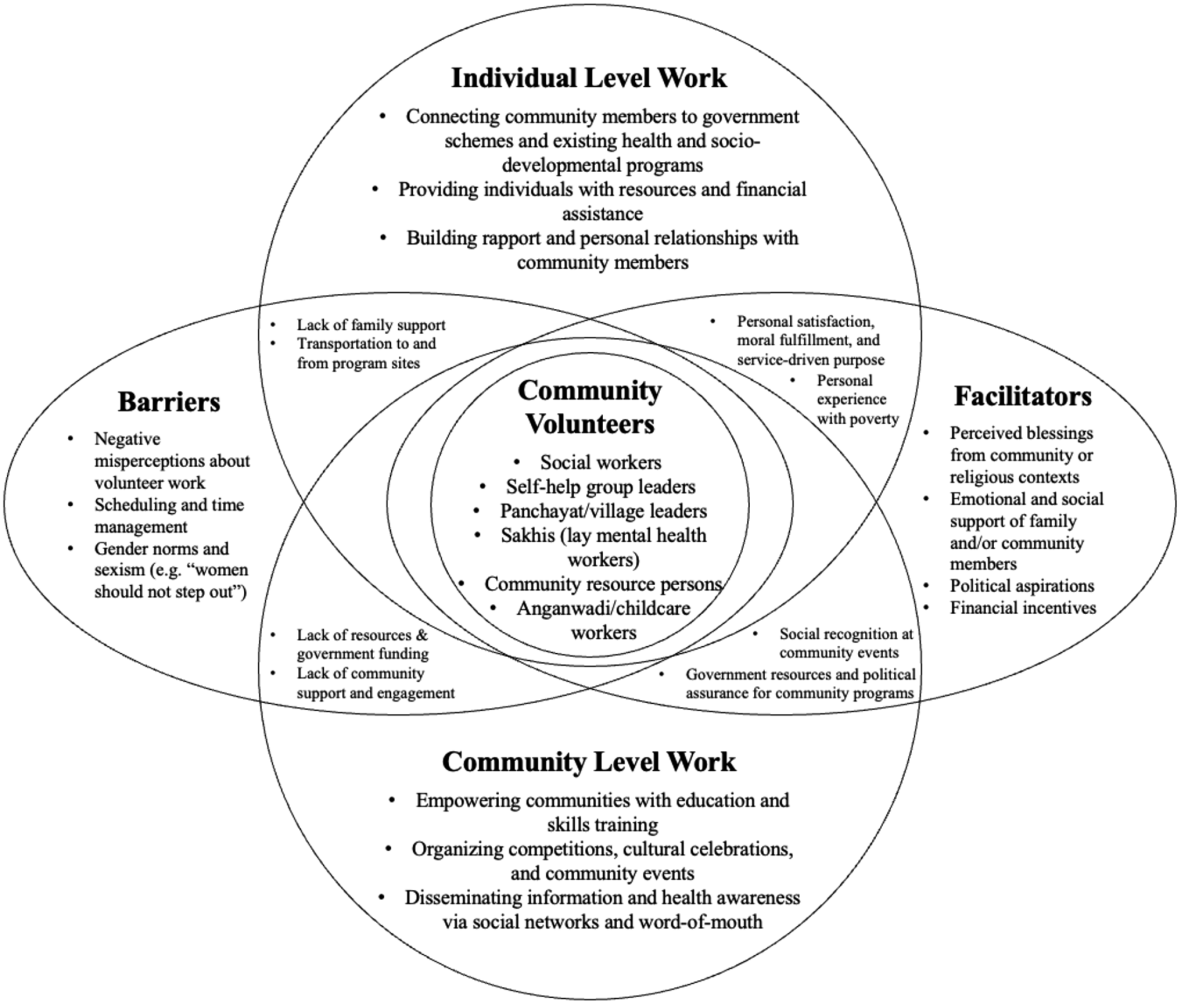
Summary of key themes and findings.

A summary of key themes identified is presented in Fig 2.

## Discussion

The objective of this study was to assess the scope of work conducted by community volunteers in Goa; barriers and facilitators for community volunteers; and strategies for integrating community engagement in demand-side interventions. Our results provide valuable insights into the experiences, motivations, and engagement strategies of community volunteers in Goa, illustrating their capacity to potentially serve as key delivery agents for demand-side mental health interventions. We report the wide-ranging scope of work, incentives, and expectations for these community volunteers, contributing to knowledge gaps on the challenges, roles, and needs of community health workers [28]. Our findings augment not only the global literature, but also shed light on the intervention development process, emphasizing the importance of formative research in identifying contextually relevant strategies for developing a potentially efficacious intervention by using the IMPRESS program as a case study [21,29]. Our study contributes to the growing body of research recognizing community health volunteers not only as treatment providers (i.e., responding to supply-side constraints), but as crucial community change agents capable of reducing demand-side barriers (e.g., by playing a key role in increasing program outreach, mental health literacy, and treatment uptake)—the often-overlooked *social* dimensions of psychosocial interventions.

Community integration, social networks, and personal relationships are crucial to volunteer work, yet community relationships remain underreported in the global literature, which often views participation and integration in local structures as complementary, but not central to the work of lay health workers [30]. The scope of work currently undertaken by our participants, whether linking community members to government schemes or organizing educational trainings, relies upon community structures. This strength in navigating and connecting individuals with available resources, alongside their role in social empowerment and skill development, makes community volunteers uniquely capable of facilitating awareness, engagement, and accompaniment to often-stigmatized mental health services. Our participants’ focus on social work and community outreach expands upon the role of community volunteers as primarily supply-side providers of psychological treatments [31], highlighting an untapped potential for generating demand and literacy for mental health treatment. Volunteer strategies incorporating influential village leaders, WhatsApp networks, and group meetings to mobilize community participation further emphasizes the necessity of collaborating with local stakeholders to leverage existing demand-side strategies.

Our findings also illustrate how broader global challenges—specifically, COVID-19—affect community volunteers and local conceptions of mental health [8,32]. Our participants reported greater awareness of the social origins of distress, particularly due to unemployment and pandemic-related hardships. Their recognition of community needs for counseling services and meditation centers illustrates the potential for community volunteers to propel mental health literacy from personal pathology to social wellbeing and collective responsibility [33]. The adaptability of volunteers, demonstrated by their transition to online platforms (e.g., WhatsApp) for training and service delivery, underscores their capacity to address global health challenges while using technology to scale up information access.

Notably, community, family, and local government support are crucial for successful interventions. Our study highlights various political, personal, and program-related barriers and facilitators that volunteers face, which are largely consistent with existing evidence [5,7]. Lack of transportation, resources, and funding for community programs in Goa reflects similar reports of economic hardships, difficult commutes, and insufficient resources for implementation of community-based programs in other settings [34–36]. Inadequate community support and participation, partially attributed to mental health stigma, poses significant barriers to volunteers, who must navigate gendered and social norms hindering help-seeking and engagement [5,37,38]. Community volunteers require the support of both their families and communities, a finding corroborated by prior studies calling for collaboration with local leaders and institutions for successful implementation [39,40].

Our study illustrates how community volunteers who have experienced upward mobility in their socio-economic statuses and those with political aspirations can be motivated to volunteer, yet inadequate social and economic support across individual, family, and institutional levels can affect this motivation. Other qualitative studies in South Asia have also reported that volunteers are motivated by community endorsement, service commitments, and social recognition (e.g., badges/certificates) [41–43]. Our results suggest that these intangible rewards are equally as important as financial incentives, adding nuance to debates concerning performance-based incentives for CHWs and community volunteers [44]. The strong emphasis on community appreciation, personal satisfaction, and spiritual blessings as motivators, alongside the fact that half of the participants sought financial compensation for their involvement in the IMPRESS intervention, points toward an interlinked incentive framework at individual, community, and health system levels [45]. Despite the tendency to focus on financial renumeration for CHWs and community volunteers [46,47], community-based interventions must also consider culturally relevant expectations, institutional support, and non-monetary benefits or relationships as key motivating factors [30,48,49].

In the global mental health space, there is growing discourse around the balance between extrinsic incentives for and intrinsic motivation of CHWs to sustain programs [50]. While trials traditionally focus on the cost-effectiveness of CHW-driven programs [51,52], our results also highlight the importance of treating volunteers as people, recognizing their agency, and integrating their motivations into intervention design. Other studies in India found similar results wherein incentives must be layered across individual (e.g., sense of identity), community (e.g., belongingness), and health system levels (e.g., adequate supervision) [53,54]. A nuanced understanding of the different forms of volunteerism can ensure program implementation aligns with community development [55], particularly in many low-resource settings increasingly dependent on community health volunteers to ensure last-mile delivery of care [56].

Given the complexity of volunteerism, we advocate for a recentering of the voices of community volunteers throughout the intervention development process. While there is growing emphasis on the importance of community engagement in enhancing the health-related outcomes of psychological interventions, most global mental health initiatives involve mid-level community engagement (e.g., community members are consulted, but have minimal influence on intervention design and implementation); the few documented community-led initiatives involved community members in leadership roles only during delivery/implementation but not design [57]. A major strength of our study is the grassroots approach to actively incorporating local knowledge from community volunteers throughout formative research, intervention design, and pre-trial implementation. This process of co-creating community engagement and involvement strategies can empower the design of equitable, people-centered, and scalable interventions [54], ensuring active participation of key stakeholders in addressing issues of implementation, sustainability, and feasibility through collective deliberation. Researchers should build upon local expertise, recognizing volunteers’ deep understanding of community dynamics and the types of services needed. Global health collaborations must also respect the subject expertise of volunteers in their community work; rather than dictating how community members should perform their existing tasks through stand-alone projects or trainings, investigators should treat them as partners through vertical integration of mental health awareness and treatment.

There are several limitations to our study. First, the sample of community volunteers interviewed was not balanced across all cadres. While we aimed for representation from various groups (social workers, panchayat members, etc.), the number of participants within each category varied, which may have influenced the breadth and depth of perspectives captured. Second, our participants may be subject to social desirability bias; their reporting of challenges, incentives, and willingness to engage with mental health interventions may be influenced by perceived expectations of the researchers, preconceptions about NGOs and government-affiliated work, and past engagements with the implementing organization’s projects. Third, this study was conducted within one state, Goa, where the unique cultural context may not be generalizable to other states in India. Fourth, we utilized only one subjective method to assess data saturation and interrater agreement, which may have biased or lead to underreporting of our results. Finally, our qualitative methods assume multiple perspectival realities that are constituted by an individual’s social context and personal history, which preclude the possibility of discovering a single, objective, external “truth” [58].

Our findings on the scope of, incentives for, and barriers/facilitators to volunteer work enabled us to identify relevant volunteer motivations and strategies that can facilitate integration of mental health into existing community work. These contextually relevant strategies have directly informed the IMPRESS intervention development and are currently being tested in the trial. A summary of key themes is presented in Table 2.

**Table 2:** Contextual summary of themes.

Theme	Contextual summary
Scope of work	**Greater emphasis on linking community members to community programs & social schemes:** the most common type of work reported by participants was actively connecting (and not just ‘referring’/sharing information) and accompanying community members (e.g., patient visits, filling out forms, etc.) to access many government schemes and health facilities. Community volunteers can play a crucial role in increasing treatment uptake, adherence, and referrals—the social dimensions of a psychosocial intervention **Social empowerment & skill development:** many volunteers interviewed were women who reported greater confidence and empowerment from their work, particularly after hosting skills & employment training for other women. Training volunteers in socially-embedded, community-based interventions can empower them to attain positive socio-developmental and health outcomes
Community engagement strategies	**Relationship-driven networking and social outreach:** participants emphasized community-based strategies for increasing program engagement and awareness through word-of-mouth, group meetings, door-to-door visits, phone call reminders, and WhatsApp networks. Volunteers leveraged influential community leaders to generate localized buy-in for program participation. A community-based approach to research must involve local stakeholders in promoting help-seeking and treatment uptake
COVID-19 and mental health awareness	**COVID-19 sparked a greater awareness of mental health in Goa:** participants reported a shift in acknowledging the social origins of distress, especially poverty and pandemic-related hardships. Community volunteers adapted to COVID-19 challenges by shifting training, social services, and group meetings to WhatsApp or online
Barriers and facilitators for community volunteers	**Community, family, and local political support is key for community-based interventions:** volunteers described poor community participation and perception of social workers as significant barriers, particularly for female leaders fighting gendered expectations. Government and family support helped volunteers secure resources, time, and space for programs
Incentives for community work	**Community recognition and social standing are equally as important as financial incentives:** almost all volunteers described community appreciation (e.g., certificates and media recognition), personal satisfaction, and spiritual blessings as powerful incentives. While many participants reported not receiving financial incentives previously, some volunteers in Goa refused to accept monetary compensation due to moral or spiritual reasons

## Conclusion

Our study provides crucial insights into the experiences, motivations, and community engagement strategies of volunteers in Goa, India, illuminating their potential to enhance the coverage and effectiveness of community-based mental health programs. Our findings highlight the existing capacity of these volunteers in demand generation, community outreach, and providing essential support, aligning with our trial’s aim to leverage community resources to augment primary care-based psychosocial treatment for depression. Future interventions and policies should prioritize the active involvement of community volunteers, building upon their local knowledge and existing networks to enhance mental health literacy, increase demand for treatment, and maximize the scalability of mental health interventions in a contextually relevant manner.
